# Similarity by state/descent and genetic vector spaces: analysis of a longitudinal family study

**DOI:** 10.1186/1471-2156-4-S1-S59

**Published:** 2003-12-31

**Authors:** Hans H Stassen, Katrin Hoffman, Christian Scharfetter

**Affiliations:** 1 Psychiatric University Hospital, Zurich, Switzerland; 2 Max Delbruck Center for Molecular Medicine, R.-Roessle-Str. 10, Berlin, Germany

## Abstract

Using the genome-wide screening data of the Framingham Heart Study (394 nuclear families, 1328 genotyped subjects, 397 marker loci) we have quantified the underlying genetic diversity through high-dimensional genetic feature vectors and constructed a genetic vector space for the analysis of population substructure. Adaptive clustering procedures led to three major subgroups that were regarded as being related to "biological" ethnicity and that included more than 70% of the subjects. Based on these subgroups we addressed the question of ethnicity-related and ethnicity-independent risk factors for coronary heart disease (CHD). To this end, we relied upon hypertension as an endophenotype of CHD and applied a multivariate sib-pair method in order to search for oligogenic marker configurations for which the sib-sib similarities deviated from the parent-offspring similarities. Indeed, the latter similarities are always "0.5" irrespective of the affection status of parents and offspring. Loci with significant contributions to the oligogenic marker configuration constituted a CHD-specific genetic vector space. We found several ethnicity-independent signals. One signal on chromosome 8 may relate to the *CYP11B1/CYP11B2 *genes.

## Background

Coronary heart disease (CHD) is one of the most common illnesses in the Western world. As with most late-onset diseases, empirical evidence from numerous studies suggests that CHD is caused by an interplay between an unspecific genetic vulnerability and environmental factors. So far, attempts to identify specific genes have not yet been successful while a series of environmental risk factors – such as overweight, cholesterol, smoking, and alcohol consumption – have been found to be associated with hypertension and cardiovascular sequelae [1,2]. Marked regional differences in the incidence of CHD may indicate a significant contribution of ethnicity-related factors to the pathogenesis of the disease [3]. All this underlines the etiologic heterogeneity and the complexity of CHD. Standard phenotype-to-genotype research strategies do not readily elucidate the genetic background of complex diseases, if 1) the contributions of single loci are small, 2) the single loci are, by themselves, neither necessary nor sufficient for developing the phenotype, 3) significant interactions between the loci are involved, and 4) there exist genetically different pathways to the phenotype in ethnically diverse populations. In contrast, the genotype-to-phenotype strategy has its main focus on oligogenic, interacting models that evaluate high-dimensional genetic feature vectors with respect to within-population and within-family similarities. This has the advantage that a population's ethnic substructure (in terms of "biological" ethnicity) can be taken into account and that multilocus variations in the genome sequence (this variation provides information on potential functional differences) can be correlated with specific quantitative conditions on the phenotype level. Consequently, the genotype-to-phenotype research strategy not only evaluates the presence or absence of the disease – as is the case with standard linkage and association methods – but also allows one to correlate the multilocus deviations in the genome sequence with quantitative scores on the phenotype level. Using the genome-wide screening data of the Framingham Heart Study [1] we have 1) quantified the underlying genetic diversity through 20-dimensional feature vectors in order to construct a genetic vector space and to analyze population substructure, 2) looked for oligogenic marker configurations for which the between-sib genetic similarity in affected and unaffected sib pairs deviated from the genetic similarity between parents and offspring, and 3) quantified the longitudinal phenotype patterns through high-dimensional feature vectors for correlations with the observed genotype structure. Our goal was to identify oligogenic configurations of risk factors that were equally valid across subpopulations and that did not depend on population substructure in terms of "biological" ethnicity.

## Methods

### Genetic vector spaces

A vector space is a well-established, universal concept for the analysis of multivariate data. *Genetic *vector spaces are spanned implicitly by a set of genetic feature vectors, where a genetic feature vector comprises a set of scalar variables. The scalar variables can include genotype measures, quantitative scores on the phenotype level, and environmental details. The intrinsic regularities inherent in a set of genetic feature vectors is revealed by systematically evaluating the distances *d*(*x*_*i*_,*x*_*j*_) between any pair of vectors *x*_*i*_,*x*_*j *_(0 ≤ *d *< ∞) or the respective similarities (similarity is inversely related to distance *s*(*x*_*i*_,*x*_*j*_) = 1/ [*d*(*x*_*i*_,*x*_*j*_) + 1] for *i, j *= 1,2,..., *n*). Similarity measures are better suited for structural analyses of empirically derived vector spaces because the similarity coefficients *s *are "normalized" such that 0 ≤ *s *≤ 1. There exists a variety of different distance and similarity measures. One distinguishes between metric and nonmetric measures depending on whether or not the "triangular" criteria

*d*(*x*_*i*_,*x*_*k*_) ≤ *d*(*x*_*i*_,*x*_*j*_) + *d*(*x*_*j*_,*x*_*k*_) and *s*(*x*_*i*_,*x*_*j*_) × *s*(*x*_*j*_,*x*_*k*_) ≤ [*s*(*x*_*i*_,*x*_*j*_) + *s*(*x*_*j*_,*x*_*k*_)] × *s*(*x*_*i*_,*x*_*k*_)

are met. In the case of metric distances the underlying vector space can be constructed from a set of vectors ("measurements") by means of a principal component analysis (PCA). The PCA axes are aligned in the direction of the vectors' largest variations identified through the largest eigenvalues of the covariance matrix. Specifically, the PCA yields a rank order of eigenvalues *e*_1 _≥ *e*_2 _≥ *e*_3 _≥ ... ≥ *e*_*n *_ ≥1 >*e*_*n*+1 _≥ ... associated with the "eigenvectors" *v*_1_, *v*_2_, *v*_3_,..., where n denotes the number of significant eigenvalues that have been extracted. The significant eigenvectors constitute the dimensionality of the vector space, whereas the vector space's orthogonal complement, associated with the insignificant eigenvalues, is eliminated. Conventionally, eigenvalues *e *≥ 1 are regarded as "significant". The amount of variance explained by the vector space can serve as a goodness-of-fit estimate.

In the case of nonmetric similarities the vector space is constructed by means of a nonmetric multidimensional scaling (NMDS) procedure [4]. This procedure relies upon the fact that under almost all circumstances a *metric *vector space can be constructed from the rank order of *nonmetric *similarities in such a way that the rank order of the resulting metric distances is identical with the rank order of the original nonmetric similarities. Of particular interest are oligogenic vector spaces spanned by n ≥ 8 uncorrelated, highly polymorphic microsatellites because such vector spaces provide a powerful means for the analysis of population admixture, thus clearing the way for a solution of the problem of genomic control [5,6].

### Genetic similarity

Central to our oligogenic approach to quantifying genetic diversity is the similarity function that enables one to quantify the genetic distances *d*(*x*_*i*_,*x*_*j*_) between feature vectors *x*_*i*_,*x*_*j *_made up by the allelic patterns of any two subjects *i*,*j *at loci *l*_1_,*l*_2_,...,*l*_*n*_. We use a nonmetric set-theoretical similarity measure that has been designed primarily to assess similarity by state (SBS) [7-10] but also it allows one to model similarity by descent (SBD) in a cross-sectional way. It is based on a step-wise mutation model of the evolution of microsatellites [11] and evaluates the fragment sizes (bp) of microsatellite alleles. Subtracting the individual offset *o *of a microsatellite from the subject's alleles a_*i *_= a_*j *_at locus, *k *the respective genotype a_*i*_a_*j *_is modeled as the rectangular area spanned by the two transformed alleles , so that the subject's feature vector at *n *loci can be regarded as an area assembled from *n *"patches". Hence, the overall similarity between the feature vectors *x*_*i*_, *x*_*j *_of two subjects *i,j *can be quantified through the set-theoretical intersection (∩: area shared by the two patterns) and the set-theoretical union (∪: total area involved)



with *w*_*k *_designating the weight of the feature vector's *k*^th ^component (proportional to its information content), and *X*._*k *_the area spanned by the two alleles *A*_*k*1_, *A*_*k*2 _of the *k*^th ^component. The specific properties of this similarity measure, regarding vector length and calibration, have been described in detail elsewhere [12]. Performance and suitability of the similarity measure have been verified through a computerized-recognition-of-person test applied to a large and representative sample of unrelated individuals. This test yielded rates of false-positive and false-negative classification errors of typically <<5% each, while the parent-offspring similarity and the within-pair similarity of sibs were 0.5.

There exist two principally different approaches to evaluating genetic similarity: 1) a set of unrelated subjects is screened for intrinsic groupings by means of genetic vector spaces and cluster analyses. This allows one to detect population stratification and to establish a concept of "biological" ethnicity when addressing the problem of genomic control. This type of analysis relates to SBS and requires a larger number of polymorphic microsatellites to recognize first-degree relatives ex nihilo. 2) The genetic similarity of a set of families is evaluated in a family-wise way (e.g., parent-offspring versus sib-sib). Several generations may be analyzed as an entity in order to optimize the performance of similarity measures or to adjust the measures in the case of population isolates. This type of analysis enables signal detection through the linkage paradigm and relates to SBD.

We have conducted computer simulations on the basis of 60 families with two affected and two unaffected offspring. The number of loci varied from 20 to 30 with an average number of 4 to 10 alleles, whereby five randomly selected loci were chosen as "affected" in terms of a 10% increase in concordance. The respective results suggested a statistical power >90% (*p *= 0.01) to detect deviations from the genetic similarity of 0.5 in affected sib pairs. The power to detect subgroups in a population was found to be of the same order of magnitude (five randomly selected loci chosen as group-specific in terms of a 10% increase of within-group concordance). Incomplete or distorted feature vectors due, for example, to small errors in allele sizes or missing alleles, have little effect on the similarity coefficients as long as the overall signal-to-noise ratio remains acceptable: e.g., if a 10% deviation in genetic similarity is to be resolved, the level of white noise caused by randomly distributed missing data must not exceed 10%.

### Adaptive clustering procedures

Using similarities *s*(*x*_*i*_,*x*_*j*_) that may depend on a specific cluster, the algorithms of adaptive clustering procedures are based on three decision regions for elements *x *and clusters *X*_*j*_



where θ ≤ 1 and *m*_*j *_denotes the center of cluster *X*_*j *_(j=1,2,...). Both constants θ and τ may be given either by a priori knowledge or must be estimated from a calibration sample. The similarity to cluster centers *m*_*j *_is often replaced by the averaged similarity



in order to derive clusters directly from similarity matrices. During cluster creation new elements are used to modify the description of established clusters, or to form centers of new clusters with prespecified initial variances, or are set aside if they fall in guard zones. The adaptive clustering procedure starts with τ chosen in such a way that each single element forms a cluster. Then τ is made successively smaller, thus allowing clusters to merge. The algorithm looks for stable solutions, i.e., for configurations of clusters where small changes of τ do not change clusters. The parameter θ defines a cluster's guard-zone, i.e., its immediate neighborhood that cannot harbor the center of another cluster. We used a 10% guard-zone (θ = 0.9) with respect to the cluster's radius.     

### Genetic diversity: univariate approach

In empirical studies the question arises as to how to construct genotypic feature vectors that constitute a problem-specific vector space. One possible approach is the use of uncorrelated, sufficiently heterogeneous microsatellites. The heterogeneity of a microsatellite (i.e., its information content and its potential contribution to oligogenic models) can be quantified through the microsatellite's multitude of different allele combinations observed in a given population. For a polymorphism with *n *alleles *a*_i _(*i *= 1,2,...,*n*) there exist *n*(*n *+ 1)/2 possible allele combinations *a*_i_*a*_j _(*i*, *j *= 1,2,...,*n*), the so-called genotypes. Defining F_(ij) _as the relative frequency [%] of the genotypes *a*_i_*a*_j _and arranging the F_(ij)_^(κ) ^(*k *= 1,2,...,*n*(*n *+ 1)/2) in descending order, such that

F_(ij)_^(1) ^≥ F_(ij)_^(2) ^≥ F_(ij)_^(3) ^≥ ... ≥ F_(ij)_^n(n+1)/2^,

we can define a heterogeneity coefficient *h *= *h*(*m*,*s*) as the number m for which the sum of the F_(ij)_^(k) ^(*k *= 1,2,...,*m*) in descending order becomes greater or equal to a prespecified percentage s, where s is typically in the range of 95–99%:



An exponential/logarithmic transformation may be used to compensate for the non-normal distribution of the heterogeneity coefficients in standard marker sets if compatible ethnicity markers have to be selected for cross-comparisons between studies.

### Data material

Our study comprised 394 nuclear families with 1328 subjects from the community-based Framingham sample. Participants aged 29 to 62 years were followed up to 52 years with up to 21 repeated assessments. Of the nuclear families, 48 included sibships with both parents, 142 with one parent, and 204 sibships without parents. On the phenotype level, blood pressure, hypertensive treatment, tobacco and alcohol consumption, total cholesterol, fasting HDL cholesterol, fasting triglycerides, and fasting glucose were examined. With respect to blood pressure, 136 sib pairs (34.5%) were concordant for normal values, 183 sib pairs (46.4%) discordant, and 75 sib pairs (19.0%) concordant for hypertension. The subjects contributed a 20-ml blood sample from which DNA was extracted and genotyped for 397 microsatellite markers (modified Weber9 marker set).

## Results

### Genetic diversity

**Figure 1:**
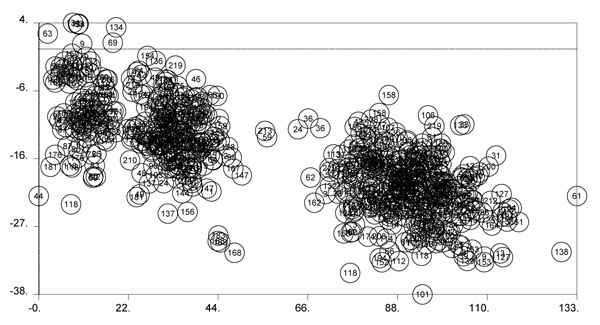
**Framingham study of hypertension: orthogonal projection onto cluster centers 1/2/3 **Structural decomposition of genetic diversity: projection of the feature vectors of 1328 subjects onto the plane defined by the three largest cluster centers.

Selecting those 20 uncorrelated markers (Table 1) that displayed the highest allelic variability *h(m,s)*, we assembled 20-dimensional feature vectors in order to derive a genetic vector space for the representation of the subjects as multidimensional points. Adaptive cluster analysis led to three major subgroups that were regarded as being related to biological ethnicity and included more than 70% of the subjects (Figure 1). Based on these subgroups we addressed the question of ethnicity-related and ethnicity-independent risk factors for CHD.

### Systematic search for oligogenic susceptibility configurations

**Figure 2:**
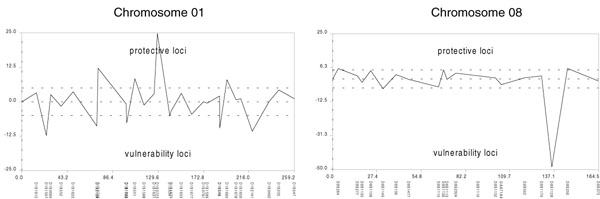
**Vulnerability-related (negative signs) and protective loci (positive signs) on chromosomes 1 and 8 as derived by the multivariate sib-pair method **The contribution of each locus to the oligogenic model of ethnicity-independent vulnerability is plotted along the y-axis (%), while the genomic regions are plotted along the x-axis (cM).

Using hypertension as endophenotype of CHD and treating the genome as a single entity, we subdivided the genetic regions, implicitly defined by the 397 marker loci, into *n *= 40 segments *s*_*i *_each including 10 markers (*i *= 1,2,...,*n*). Each segment *s*_*i *_was systematically combined with each segment *s*_*j*_, thus yielding *n*(*n *- 1)/2 feature vectors of length 20 that allowed us to detect interactions between any two marker loci (*i, j *= 1,2,...,n; *j *>*i*). Interactions were deemed to be present if the joint effect of two markers deviated from the sum of their single effects. We then determined the distribution of parent-offspring similarities together with the distribution of the between-sib similarities of affected, unaffected, and discordant sib pairs. Subsequently, our signal detection algorithm looked for significant differences between the parent-offspring similarities and the sib-sib similarities whose mean value was expected to deviate in affected sib pairs from the parent-offspring value if the feature vector included markers close to vulnerability or protection genes. Those loci that contributed significantly to deviations in the expected values were included in the oligogenic configuration that constituted our CHD-specific genetic vector space. We found several ethnicity-independent signals. One signal on chromosome 8 (Figure2) may relate to the *CYP11B1/CYP11B2 *genes at 142.3 cM (11β-hydroxylase, aldosterone-synthase).

## Discussion

Our quantitative concept of vulnerability and protection factors assumes etiologic and phenotypic heterogeneity in such a way that only a certain proportion of the affected sib pairs exhibit an elevated SBD/SBS score at a certain locus within an oligogenic configuration. Thus, each locus of the configuration is regarded as being, by itself, neither necessary nor sufficient for developing the phenotype. There also exist subsets of affected sib pairs with a significant genetic dissimilarity at a locus of the configuration as well. Since a "dissimilarity locus" interacts with at least one of the vulnerability loci, it is likely that it modifies the genetic risk of the phenotype. We therefore conjecture that affected siblings who are dissimilar on the genotype level also exhibit differences on the phenotype level, perhaps, in terms of onset and severity of illness.

As to the genetic analysis of complex traits, oligogenic approaches to quantifying genetic diversity complement standard linkage and association methods by following a genotype-to-phenotype research strategy. This has the advantage that multilocus variations in the genome sequence can be correlated with specific quantitative conditions on the phenotype level, that is, not only with the presence or absence of the disease but also with lifestyle and environmental details. Such a viewpoint appears to be of particular importance in the case of CHD, where environmental factors such as smoking, alcohol consumption, obesity, and comorbid personality traits modify both the risk of and the prognosis for the disease.

## Figures and Tables

**Table 1:** Structural decomposition of genetic diversity. Those 20 markers that displayed the highest allelic variability in the sample and had acceptable missing data rates were selected for the construction of genetic feature vectors.

**Polymorphic markers used to quantify biological ethnicity**				
**Marker**	**Missing**	**Location cM**	**Nucleotide**	Heterogeneity

D1S1612	4%	13.8	4	17.6
D2S2976	7%	3.0	4	27.0
D2S1360	5%	40.0	4	20.2
D2S1788	1%	61.5	4	36.0
D3S1259	5%	28.2	2	17.6
D3S2427	4%	165.7	4	27.0
D4S2632	2%	54.2	4	21.2
D6S305	4%	161.5	2	19.4
D7S513	4%	22.6	2	43.6
D7S2204	3%	87.1	4	22.1
D7S1804	5%	126.0	4	26.0
D7S2195	4%	139.0	4	19.4
D8S277	3%	15.2	2	22.1
D11S1986	3%	98.8	4	50.4
D12S391	5%	27.9	4	21.2
D13S788	6%	54.8	4	18.5
D15S822	7%	16.9	4	36.0
D17S928	8%	135.7	2	21.2
D20S470	2%	44.6	4	16.8
D21S2055	6%	50.7	4	64.0
